# Retention of Pediatric BioFlx Crowns Versus Stainless Steel Crowns Using Different Types of Luting Cements: An In Vitro Study

**DOI:** 10.3390/ma18061287

**Published:** 2025-03-14

**Authors:** Amjad A. Al Mawash, Ayman M. Sulimany, Latifa A. Alhowaish, Abdullah S. Alayad, Omar A. Bawazir

**Affiliations:** 1 Department of Pediatric Dentistry and Orthodontics, College of Dentistry, King Saud University, Riyadh 12372, Saudi Arabia; asulimany@ksu.edu.sa (A.M.S.); lalhowaish@ksu.edu.sa (L.A.A.); obawazir@ksu.edu.sa (O.A.B.); 2 Department of Restorative Dental Science, College of Dentistry, King Saud University, Riyadh 12372, Saudi Arabia; alayad@ksu.edu.sa

**Keywords:** BioFlx crowns, dental cements, retention, stainless steel crown

## Abstract

BioFlx crowns (BFCs) have been introduced in the dental market, combining the flexibility of stainless steel crowns (SSCs) with the esthetic appeal of preformed zirconia crowns. However, the existing literature does not provide adequate insights regarding the retentive strength of various types of luting cement with these newly developed BFCs. Therefore, this study aimed to evaluate and compare the retentive strength of BFCs and SSCs with different types of luting cement (glass ionomer cement [GIC], resin-modified glass ionomer cement [RMGIC], self-adhesive resin cement [SARC], and polycarboxylate cement [PXC]). A total of 160 standardized resin dies were fabricated and divided into two groups based on the type of crown (BFCs or SSCs). Each group was further subdivided into four subgroups (20/group) based on the luting cement used for cementing the crown on the die. A pullout test was performed using a universal testing machine to measure the retentive strength required for crown dislodgement. The residual cement in the crown was scored to determine the cement failure pattern. Data were analyzed using two-way analyses of variance (ANOVAs) to evaluate the interaction between the cement and the type of crown on retentive strength, followed by an independent *t*-test. Furthermore, Welch’s ANOVA and Dunnett’s T3 test were used to assess the impact of various types of luting cement on the retentive strength of each crown. The CFP was assessed by comparing the scores using descriptive statistics. Statistical significance was set at *p* < 0.05. The mean retentive strength of SSCs and BFCs was the highest with SARC (560.29 ± 8.74 N; 657.72 ± 20.60 N), followed by RMGIC (534.20 ± 22.84 N; 454.90 ± 7.95 N) and GIC (435.14 ± 8.66 N; 237.68 ± 9.37 N), while the lowest was with PXC (365.67 ± 19.11 N; 131.26 ± 5.37 N). A significant difference in retention was observed between the crowns (*p* < 0.05). Cement failure primarily manifested as adhesive failures in the SARC and RMGIC groups; however, both adhesive and cohesive failures occurred in the GIC and PXC groups. Thus, SSCs demonstrate significantly higher retention than BFCs across all types of luting cements, except when using SARC. Within the limitations of this in vitro study, SSCs emerge as the preferred choice for full-coverage restorations that require optimal retention and durability. Nevertheless, BFCs with SARC provide a viable alternative when esthetic considerations are prioritized.

## 1. Introduction

Primary dentition is crucial for maintaining space in the dental arch for the eruption of permanent teeth and plays a significant role in phonetics, esthetics, and mastication [1]. Dental caries is the predominant cause of premature loss of primary teeth [2]. A variety of materials have been used to manage carious primary teeth effectively, including amalgam, glass ionomer cement, resin-modified glass ionomer cements, compomers, composite resin, and preformed crowns, each showing different rates of success [3]. The American Academy of Pediatric Dentistry (AAPD) recommends full-coverage restorations or preformed crowns for primary molars with extensive carious lesions, multiple affected surfaces, cervical decalcification, or following the pulpal therapy [4]. They are also advisable for primary molars intended as abutments for space maintainers, for intermediate restoration of fractured teeth, and for definitive treatment in cases with a high risk of caries. This treatment option provides durability and long-lasting protection, and prevents recurrence of caries [5,6].

Preformed metal crowns, commonly referred to as stainless steel crowns (SSCs), are considered the gold standard in pediatric restorative dentistry due to their affordability, adaptability, minimal tooth reduction, and superior retention [7]. Their proven durability and long-term clinical success make them a highly reliable choice for restoring primary teeth [8]. Pediatric dentists have widely used SSCs for several decades to restore severely carious primary molars [9,10]. Although these crowns have several advantages, they have certain limitations, primarily their metallic appearance, which lacks esthetic appeal. The esthetic concerns associated with SSCs can significantly impact both the child’s self-esteem and parental satisfaction, thus making them an important consideration in treatment planning [11,12]. Consequently, several esthetic alternatives to SSCs have been developed, including pre-veneered and open-faced SSCs. Although these choices enhance esthetic appeal, they also pose significant drawbacks, such as compromised gingival health, longer appointment durations, restricted contouring and crimping, and the possibility of veneer resin chipping or fracturing [10,13,14]. Hence, practitioners have sought a crown that combines the durability and longevity of SSC with an esthetic appeal. In 2008, pediatric zirconia crowns (PZCs) were introduced as esthetically full-coverage restorations exhibiting superior mechanical properties [15]. However, PZCs require more tooth reduction than SSCs, which can extend the crown preparation and fitting process, particularly for pediatric patients. Additionally, their high cost makes them financially unaffordable for many patients [16]. More recently, BioFlx crowns have been introduced in the dental market as a notable advancement in pediatric dentistry. The manufacturers of these crowns claim that they are combining the flexibility of SSCs with the esthetic appeal of PZCs. They are biocompatible, flexible, and constructed from high-impact, high-strength hybrid resin polymers that are molded to fit the teeth actively [17].

Although preformed pediatric crowns are extensively utilized in pediatric dentistry, they are prone to failure due to crown loss resulting from cement failure [18]. The retention of crowns plays a critical role in maintaining the function and integrity of primary molars, ensuring proper mastication, speech development, and space maintenance for the eruption of permanent teeth [4,19]. Properly retained crowns significantly contribute to the prevention of complications, including tooth loss, infection, and misalignment, all of which can adversely affect a child’s oral health and overall development [3,20]. Crown loss typically occurs when the bond between the crown and the underlying tooth is compromised, often due to cement failure. As a result, selecting and applying appropriate luting cement is a fundamental aspect of ensuring crown stability and longevity, as the retention of crowns primarily relies on the type of luting cement [21,22,23,24].

Luting cement acts as the adhesive that fills the gap and secures the crown to the underlying tooth structure. Selecting the appropriate type of luting cement is crucial for preventing crown dislodgement and minimizing the risk of crown failure [25,26]. The cement must meet several key requirements to ensure long-term success. It should ensure strong adhesion to both the tooth and crown material, along with high tensile and compressive strength, to withstand chewing forces and prevent premature crown loss. Additionally, it must have biocompatibility with both the tooth and the crown to ensure a secure fit and maintain an effective marginal seal, which helps prevent microleakage. It should also demonstrate low solubility in oral fluids, minimal film thickness for optimal crown adaptation, low viscosity for easy application, and adequate working time with a quick setting process [27,28].

In this context, several laboratory and clinical research studies have investigated the retention strength of different types of crowns, such as SSCs and PZCs, when cemented with various luting cements, including glass ionomer cement (GIC), resin-modified glass ionomer cement (RMGIC), self-adhesive resin cement (SARC), zinc polycarboxylate cement (PXC), and zinc phosphate cement (ZPC) [27,29,30,31,32]. These studies highlight how the choice of luting cement affects crown retention, with each type of cement showing different levels of effectiveness in securing the crown to the underlying tooth structure.

However, the existing literature is insufficient in providing a clue regarding the retentive strength of various types of luting cement used with newly developed BFCs. Therefore, this study aimed to evaluate the retentive strength of BFCs with the most used types of cement in clinical settings and previous studies (GIC, RMGIC, SARC, and PXC) compared to SSCs.

The null hypotheses are as follows: (1) there is no significant interaction between the different types of crowns and different types of luting cement; (2) there is no significant difference between the different types of crowns in terms of retentive strength; (3) there is no significant effect of different types of luting cement on the retentive strength within each crown.

## 2. Materials and Methods

### 2.1. Die Preparation

**Figure 1:**
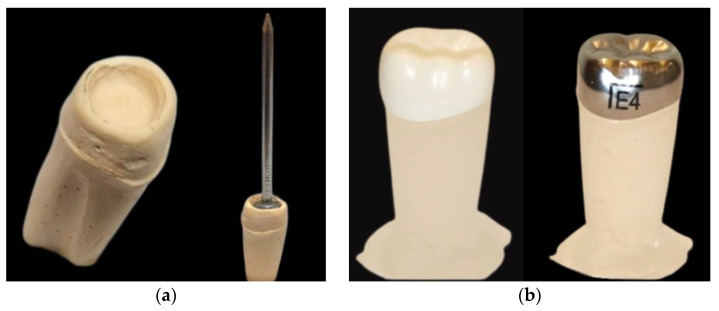
(**a**) A stone die was deepened by carving the occlusal surface to accommodate the nail head. (**b**) Crowns were tried on resin dies to ensure a proper fit.

A total of 160 resin dies were fabricated through the following procedure. First, the mandibular left second primary molar NuSmile^®^ BioFlx^®^ crown (BFC) (size 4; NuSmile Inc., Houston, TX, USA) was attached to the silicone-preformed mold, and the inner surface of the crown was separated using petroleum jelly (Vaseline) as a separating medium. Subsequently, a type IV dental die stone (GC Fujirock^®^ EP, Leuven, Belgium) was poured into the BFC and allowed to set. Once the dental stone was set, the crown was removed from the stone die, and excess material was removed. Afterward, the stone die’s occlusal surface was deepened by carving to facilitate the placement of the nail head (Figure 1a). A silicone mold was made from this stone die, which is a negative replica of the crown, using Deguform^®^ Plus (Dentsply International, Hanau, Germany) via the impression process. Subsequently, the resin dies were created from this silicone mold using Interacrylic Ortho Resin (Interacrylic Ortho, Interdent, Celje, Slovenia) and allowed to set for 24 h. Following that, the BFCs were tried on the resin dies to ensure a proper fit, and any excess material was removed with a finishing composite bur. The same resin dies of BFCs were utilized for the mandibular left second primary molars 3M™ ESPE SSCs (size 4; 3M™ ESPE, St. Paul, MN, USA), as the SSC was fitted precisely into the die (Figure 1b).

### 2.2. Crown Preparation

**Figure 2:**
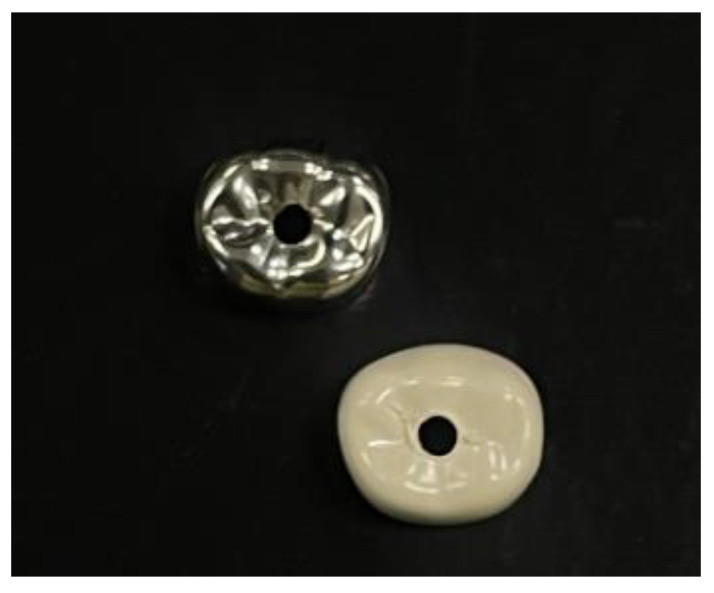
Preparation of a hole in the central fossa of the crowns.

For all the crowns (BFCs and SSCs), a diamond round bur (FD-DS0447; Diaswiss SA, Nyon, Switzerland) with a head diameter of 1 mm was used to drill the central fossa (Figure 2). A nail with the following dimensions was inserted through the crown’s aperture: 25 mm in height, 1 mm in shank diameter, and 2.5 mm in flattened head diameter. This procedure enabled easy attachment of the specimens to the test machine.

### 2.3. Cementation

The standardized resin dies of each type of crown (80/group) were randomly allocated to one of the four experimental groups according to the type of luting cement used (20/group): Group I, GIC (3M™ Ketac™ Cem Aplicap™ 3M ESPE, Seefeld, Germany); Group II, RMGIC (RelyX^™^ Luting 2; 3M ESPE, St. Paul, MN, USA); Group III, SARC (RelyX™ U200; 3M ESPE, Seefeld, Germany); and Group IV, PXC (Poly-F^®^; Dentsply, Konstanz, Germany) (Figure S1). All the crowns were cemented according to the manufacturer’s instructions by a single trained pediatric dentist, who was trained during a pilot study to minimize variability. The luting cement was loaded onto two-thirds of the inner surface of each crown. Subsequently, the crown was correctly placed on the resin die using finger pressure, and an axial weight of 5 kg was applied to the crowns using a loading apparatus for 10 min to maintain the stability of the crowns during the cement setting process [33] (Figure S2a,b). Excess cement was removed from the cervical margin of the crown using a sharp-spoon excavator. The same protocol was applied to all the specimens. The crowns cemented with RMGIC and SARC were light-cured for 20 s on each side using a single-wave light-cure unit with a light intensity of 1200 mW/cm^2^ (Elipar S10, 3M™ ESPE™, St. Paul, MN, USA). Following that, all the specimens were immersed in distilled water and placed in an incubator (GI2 So-Low, Cincinnati, OH, USA) at 37 °C and 100% humidity for 24 h to ensure a complete set of the cement [23].

### 2.4. Retentive Strength Measurement

**Figure 3:**
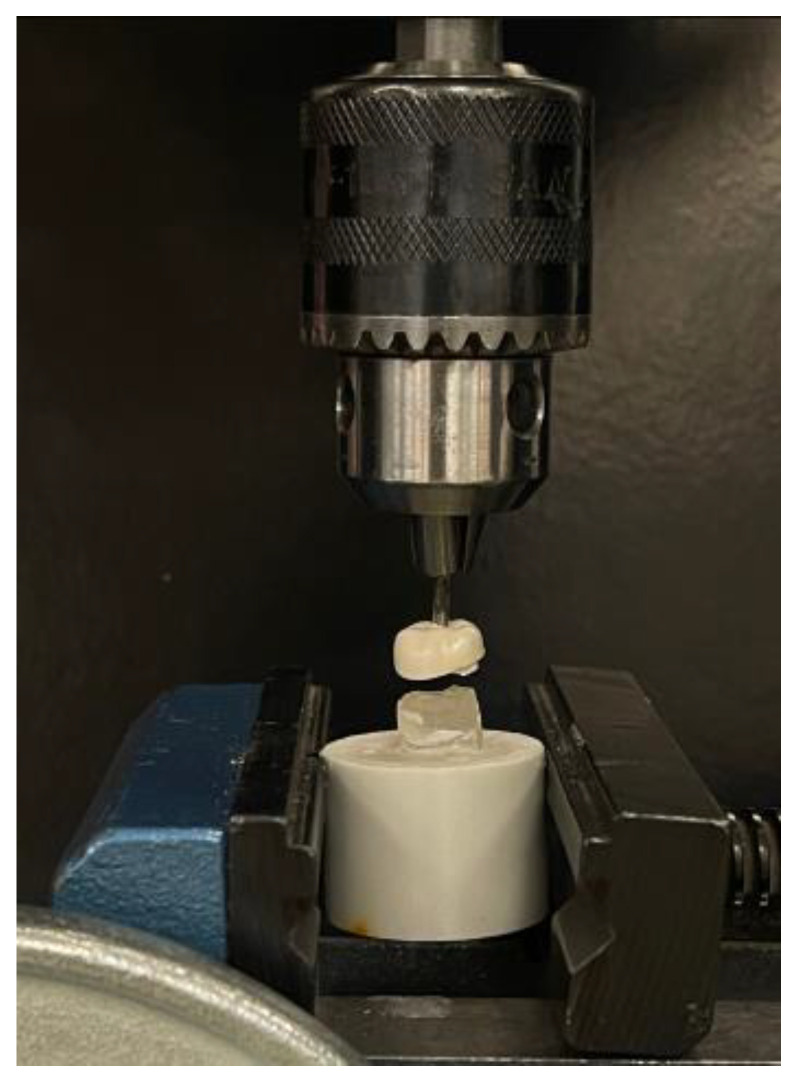
BioFlx crown during pullout test.

The retention strengths of the crowns were measured using a universal testing machine (Instron, model no. 8500; Illinois Tool Works, Inc., Norwood, MA, USA). The specimen was stabilized on the machine by directly engaging it with the lower crosshead, and the nail was positioned between the two surfaces of the upper crosshead. In the pullout test, the load was applied parallel to the long axis of the crown at a speed of 0.5 mm/min [34]. Load application was gradually increased from zero until the cemented crown was completely detached from the resin die; the retentive force was recorded in Newtons (N) (Figure 3). All the specimens were processed in the same manner.

### 2.5. Assessment of Cement Failure Pattern

Following the completion of the pullout test, specimens were visually assessed for cement failure patterns (CFPs) (Figure S2c) and scored according to Jing et al. (2019) (Table 1) [35].

### 2.6. Sample Size Calculation and Statistical Analysis

The G*Power 3.1.9.4 software (Heinrich-Heine-Universität Düsseldorf, Düsseldorf, Germany) was used to determine the required sample size; a minimum of 160 crowns were required (80/group) based on an effect size of 0.38 and a power of 0.9 at α = 0.05. Data were analyzed using the SPSS software version 24 (IBM Inc., Chicago, IL, USA). Two-way analysis of variance (ANOVA) was used to evaluate the interaction between the cement and the type of crown on the retentive strength, followed by an independent *t*-test. Furthermore, a one-way ANOVA was used to assess the impact of various types of luting cement on the retentive strength of each crown. First, the normality of the data distribution was verified using the Shapiro–Wilk test; no significant evidence of non-normality was found (*p* > 0.05). However, Levene’s test resulted in significant evidence that the assumption of homogeneity of variances was violated (*p* < 0.00). Thus, Welch’s ANOVA and Dunnett’s post hoc tests were employed to evaluate the cement within each crown group. Statistical significance was set at *p* < 0.05. The CFP was assessed by comparing the scores using descriptive statistics.

## 3. Results

**Figure 4:**
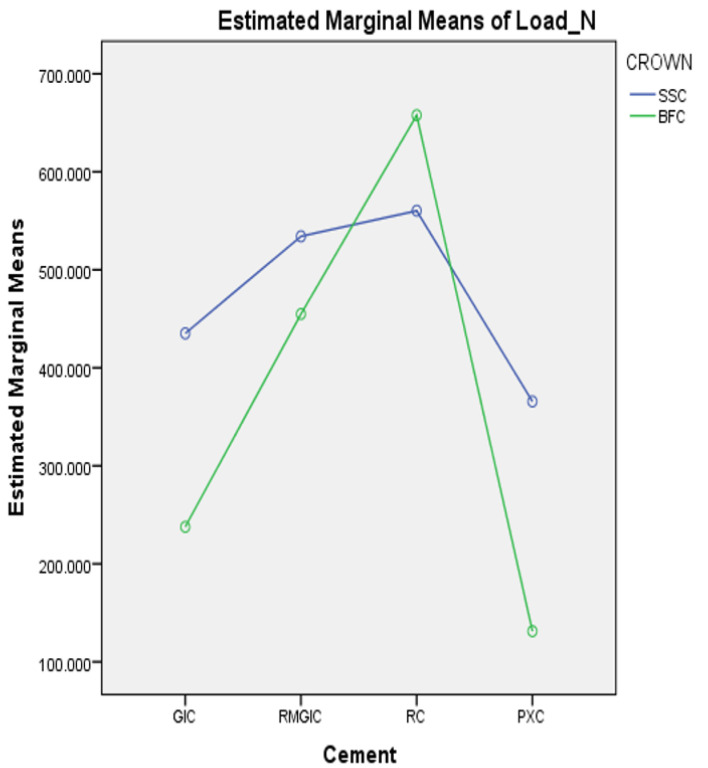
Interaction between the types of luting cement and crown materials in relation to retentive strength.

Two-way ANOVA demonstrated a significant interaction between the types of luting cement and crowns regarding the retentive strength (*p* < 0.05) (Figure 4).

**Figure 5:**
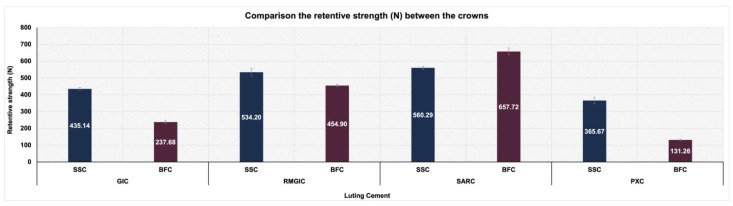
Comparison of the retentive strength (N) between SSCs and BFCs within each cement.

The SSCs exhibited significantly greater retention strength than BFCs across all cements, except in the SARC group, where BFCs demonstrated superior strength (*p* < 0.05) (Table 2; Figure 5).

Table 3 lists the effects of various luting cements on the retentive strength of each crown. A significant difference was observed in the retentive strength between all types of luting cements for the same type of crown (*p* < 0.05). The SARC exhibited the maximum retentive strength (560.29 ± 8.74 N; 657.72 ± 20.60 N), followed by RMGIC (534.20 ± 22.84 N; 454.90 ± 7.95 N), GIC (435.14 ± 8.66 N; 237.68 ± 9.37 N), and PXC (365.67 ± 19.11 N; 131.26 ± 5.37 N), for both SSCs and BFCs, respectively.

The CFP scores, including counts and percentages, are presented in Table 4. In the SSC group, a score of 1 was predominant in the SARC, RMGIC, and GIC groups (90%, 85%, and 65%, respectively), while a score of 4 was most common in the PXC group (75%). Conversely, the BFC group exhibited a more frequent score of 4 in the SARC and RMGIC groups (90% and 65%, respectively), whereas scores of 3 and 2 were more prevalent in the GIC and PXC groups (80% and 80%, respectively).

## 4. Discussion

The crown’s retention is a crucial element in assessing crown efficacy in primary teeth. It is achieved through both mechanical and chemical means. An appropriate size of crowns, contouring, and crimping provides mechanical retention, whereas chemical retention is achieved using a luting cement that seals the gap between the crown and abutment surfaces and maintains the crown until the time of exfoliation [16,33,36,37,38,39]. The choice of luting cement directly impacts the retention and longevity of preformed crowns. Various types of luting cements are utilized for the cementation of crowns. Over the past century, ZPCs have been commonly utilized for crown cementation. However, they lack chemical adhesion to tooth structure and depend entirely on mechanical retention, which leads to reduced adhesive strength. Despite having high compressive strength, they can be potentially harmful to vital pulp tissue because of their low pH [23]. Therefore, PXCs have been introduced as the cement that chemically adheres to enamel and dentin. They exhibit a greater adhesive strength compared to ZPCs and are compatible with vital tissue. However, they undergo significant plastic deformation when subjected to masticatory forces, and their high viscosity and low resistance to degradation in the oral cavity limit their application [23,40]. For that reason, GICs were introduced, and it was found that this type of cement can adhere to enamel and metal surfaces and is extensively preferred due to its ability to release fluoride, which assists in the prevention of recurrent caries [41]. Nevertheless, GICs possess certain limitations, such as an extended maturation period and susceptibility to moisture during the initial setting phase [42]. To overcome these shortcomings, RMGICs have been developed, effectively integrating the advantageous properties of traditional GICs, including fluoride release and adhesion, with improved physical properties, thus minimizing the likelihood of cohesive failure [43]. Recently, resin cements have become increasingly popular, typically using an etch-and-rinse or a self-etch adhesive system along with low-viscosity, dual-polymerizing resin cement. Nevertheless, the multi-step bonding process associated with these systems can be intricate, necessitating meticulous technique and considerable time. To simplify the complexities inherent to this process, a novel generation of self-adhesive resin cement has been introduced. These advanced cements eliminate the requirement for separate etching, priming, and bonding phases by integrating acidic monomers. The multifunctional phosphate-based acidic methacrylates present in these formulations are capable of reacting with the basic fillers within the luting cement, as well as with the hydroxyapatite found in hard dental tissue [44]. Recently, BFCs have been introduced as tooth-colored full-coverage restorations. However, there are limited data regarding the retentive strength of these crowns with various types of luting cement. Therefore, it is essential to assess the performance of common types of luting cement used in clinical settings with these new crowns and to compare them to the gold standard (SSCs).

Based on the results obtained in this study, the first and second null hypotheses were rejected, as there is a significant interaction between the types of luting cement and the retention strength of crowns. The SSCs were significantly superior to BFCs across all types of luting cement, except for SARC, where BFCs demonstrated superiority. These findings align with those of a randomized controlled study conducted by Agrawal et al. (2022), which demonstrated that 86.67% of PZCs and 100% of SSCs that were cemented with type I GIC were retained at 3 months following the restoration of primary molars [10]. Moreover, this corresponds with another clinical study conducted by Taran et al. (2018) that assessed the clinical success and periodontal health of primary molars restored with SSCs or PZCs cemented with GIC after 12 months. In their study, 2 out of 15 PZCs were lost within one month of placement, whereas all SSCs were retained for 12 months [45]. Kayal et al. (2023) conducted an ex-vivo study comparing the retention strength of SSCs and two types of PZCs cemented using GIC and found that the retention strength of SSCs was considerably greater than that of PZCs; however, no significant difference was observed between the two types of PZCs [46]. In addition, these results are consistent with those reported by Mandyala et al. (2024), who compared fiberglass (Figaro) crowns with SSCs using GIC and RMGIC and found that SSCs exhibited greater retention strength [47]. These findings are comparable to those of the present study, which demonstrated that SSCs exhibited superior retention to BFCs. This could be attributed to the snug fit of the SSC margin to the tooth surfaces, particularly in the undercut areas of the prepared tooth [41], as SSCs exhibit superior marginal sealing to BFCs [17] and other esthetic crowns [48,49] (Table S2).

Furthermore, the third null hypothesis was rejected, as the findings of the current study demonstrated a significant difference in the retentive strength among the different types of luting cement used with each crown. SARC exhibited the highest retentive strength, followed by RMGIC and GIC, whereas PXC demonstrated the lowest values for both crowns. This finding is consistent with those reported in previous in vitro studies, demonstrating the superiority of SARC to RMGIC [31,42,50] and GIC [18,27,42,50] in retaining SSCs on extracted teeth. The SARC exhibits superior properties, including enhanced strength, low film thickness, and extremely low solubility in oral settings [50]. It also contributes to both micromechanical retention and chemical interactions between the acidic phosphate groups of the monomer and the dentin/enamel (abutment surfaces) [51]. Consequently, these findings elucidate the reason for the superior retentive strength of SARC, which is attributable to the formation of polymer–polymer-linked connections between the cement and BFCs, which consist of hybrid resin polymer materials. The hybrid matrix reacts with the monomeric acidic groups to chemically and mechanically interlock the crown and cement. In the present study, RelyX™ Luting 2 and RelyX™ U200 were used for cementation. RelyX™ Luting 2 is a self-cure RMGIC; however, the manufacturer recommends optional light-curing to accelerate the setting process. RelyX™ U200, a dual-cure SARC, was selected for its ability to simplify the procedure by eliminating the need for substrate pretreatment. Both cements are suggested for the permanent cementation of metal and ceramic crowns. In this study, specimens were allowed to set for 10 min under axial force application to ensure proper adaptation. Traditional polymerization protocols were adhered to according to the manufacturer’s instructions to achieve complete cement polymerization and optimize the bonding performance.

Moreover, the better performance of RMGIC in comparison to GIC obtained in this study is consistent with the findings of other comparative in vitro studies that evaluated the retention of SSCs on primary molars using GIC and RMGIC [18,50,52]. Similarly, Walia et al. (2021) evaluated the retention of four different types of PZCs cemented on composite dies using GIC and two distinct RMGIC types and found that RMGICs considerably outperformed GIC in terms of crown retention [32]. Mandyala et al. (2024) reported that the retention of fiberglass (Figaro) crowns cemented on extracted primary molars with RMGIC was higher than that with GIC [47]. The RMGIC showed superior mechanical and physical properties than conventional GIC, including a lower modulus of elasticity and higher cohesive strength [53]. Additionally, the bond strength of RMGIC to tooth [54] and resin composites [55,56] is superior to that of conventional GIC. Furthermore, the hybrid resin polymer of the crown and the HEMA polymers in the cement may contribute to the mechanical interlocking between the RMGIC and BFC surfaces.

Additionally, the study findings align with those of other in vitro studies that evaluated and compared the retentive strength of SSCs cemented to natural teeth using PXC in comparison to GIC [23,27,30,50], RMGIC [50], and SARC [27,50]; SSCs cemented with PXC demonstrated markedly lower retention than the other cements. Generally, PXC adheres mainly to enamel; however, weaker adhesion to dentin occurs owing to the chelation reaction between the carboxyl groups and calcium in the tooth structure [57]. Conversely, GIC formed stronger bonds with the tooth structure and stainless steel than PXC [30]. Further, PXC possesses significantly higher plastic deformation, making it unsuitable for use in regions areas with high masticatory stress [23,40]. Therefore, the low retentive strength of PXC in this study can be explained by its fragility resulting from its thin-film thickness, chemical bonding, and plastic deformation.

However, in contrast to this sequence of luting cement performance, several laboratory and clinical studies have demonstrated that PZCs cemented with GIC display significantly higher retention than those cemented with RMGIC [34,58,59] and SARC [34]. This may be due to the subgingival tooth preparation for PZCs, which frequently leads to gingival hemorrhage and insufficient moisture control. Additionally, several in vitro studies have demonstrated that SSCs cemented with GIC exhibit greater retention than those cemented with RMGIC [42,47,60]. These differences may be attributed to the use of different techniques and various cement brands or noncompliance with manufacturing instructions.

As a part of this study, the CFP was assessed after crown detachment for four cement types. Based on the scores, cement failure can occur at the cement–crown interface (CFP score 1; adhesive failure), where the highest amount of cement remains on the die surface; at the die–cement interface (CFP score 4; adhesive failure), where the highest amount of residual cement is present on the internal surface of the crown; or at both interfaces (CFP scores 2 and 3; cohesive failure), where less than 75% of the cement is retained in either the die or crown. Among the SSCs, adhesive failure was predominant (occurring at the cement–crown interface [CFP score 1]) in the GIC, RMGIC, and SARC groups, whereas in the PXC group, it was observed at the die–cement interface (CFP score 4). These findings are comparable to those by Noffsinger et al. (1983), who reported cement–crown interface failure (adhesive) when GIC was used to cement the SSCs on natural teeth [23]. However, this contradicts the findings of Kameli et al. (2021), who used the same types of luting cement (GIC, RMGIC, SARC, and PXC) to cement the SSCs on primary teeth and demonstrated that failures predominantly occurred at both interfaces (cohesive failure) across all cement types [50]. Kayal et al. (2023) reported cement failure at the tooth–cement interface in the SSCs cemented on primary molars using GIC [46]. Discrepancies in failure patterns may result from various factors including the abutment surface, different scoring indices, crosshead speeds, and crown detachment techniques. The failure patterns of BFCs were primarily characterized by adhesive failure (CFP score 4) in RMGIC and SARC, which is associated with the majority adhering to the crown (adhesive failure). In contrast, cohesive failure was observed in GIC and PXC (CFP scores of 3 and 2, respectively); the cement primarily adhered to both the die and the crown. These findings align with those by Walia et al. (2021), who evaluated the failure modes of GIC and RMGIC for cementing other esthetic crowns (PZCs) on composite dies. The GIC predominantly demonstrated cohesive failure, whereas RMGIC exhibited adhesive failure at the die–cement interface [32]. Furthermore, the low retention of GIC and PXC may be attributed to the spontaneous cohesive fracture of the cement, the tension generated by contraction during the setting process, and low tensile strength and fracture toughness [61].

Currently, to the best of our knowledge, only one clinical trial conducted by Patil et al. (2024) has demonstrated that 96% of SSCs and 89.35% of BFCs cemented with type I GIC are retained for 12 months after restoring the primary molars [62]. This in vitro study provides baseline information regarding the retentive strength of BFCs with four different types of luting cements. Additionally, the CFP following detachment of the crowns was examined. Further details about all the materials used in this study are available in Table S2.

Nonetheless, this study has certain limitations that must be acknowledged. The present study is a laboratory study that lacks long-term follow-up and used resin die model replicas instead of primary teeth to standardize the specimens, which would have been challenging with extracted primary teeth. Moreover, it is important to recognize that the results from this study cannot be compared to those from other investigations on natural teeth, as cement may behave differently depending on the abutment surface. Furthermore, the clinical settings, including physiological and dynamic factors, have not been accurately replicated. It would be ideal to conduct a prospective clinical study to compare the retentive strength of BFCs using a variety of luting cements and evaluate the long-term performance of these crowns under physiological and dynamic conditions, including factors such as mastication forces, wear, moisture exposure, and oral pH and temperature. In addition, saliva contamination and gingival bleeding at the time of cementation, as well as oral habits and brushing, were evaluated. All these factors may influence the clinical retentive strength of crowns.

## 5. Conclusions

Considering the limitations of the present in vitro study, the following conclusions were drawn:

1. Across all types of cement, SSCs exhibited significantly greater retention strengths than BFCs, except for SARC, where BFCs exhibited superiority.
2. Among the four types of luting cement examined, SARC showed the highest retentive strength, whereas PXC demonstrated the lowest.
3. Adhesive failure was the predominant failure pattern in SSCs; however, both adhesive and cohesive failures were observed in BFCs.

## Figures and Tables

**Table 1:** Categorization of cement failure pattern.

Score	Definition
1	More than 75% of cement left on the resin die.
2	Between 50 and 75% of cement left on the resin die.
3	Between 25 and 50% of cement left on the resin die.
4	More than 75% of cement left on the crown.

**Table 2:** Comparison of the retentive strength (N) between SSCs and BFCs within each cement.

	GIC	RMGIC	SARC	PXC
SSC	435.14 ± 8.66	534.20 ± 22.84	560.29 ± 8.74	365.67 ± 19.11
BFC	237.68 ± 9.37	454.90 ± 7.95	657.72 ± 20.60	131.26 ± 5.37
*p*-Value *	<0.001	<0.001	<0.001	<0.001

Abbreviations used in this table: SSC = stainless steel crown; BFC = BioFlx crown; GIC = glass ionomer cement; RMGIC = resin-modified glass ionomer cement; SARC = self-adhesive resin cement; PXC = zinc polycarboxylate cement. * An-independent t-test showed statistically significant differences between the groups (*p*-value < 0.05).

**Table 3:** Distribution and comparison of mean retentive strength values (N) across all groups.

Crown	Cement	n	Retentive Strength (N)	95% Confidence Interval for Mean		*p*-Value *
				Lower	Upper	
SSC	GIC	20	435.14 ± 8.66 ^b^	431.08	439.19	<0.001
	RMGIC	20	534.20 ± 22.84 ^c^	523.52	544.89	
	SARC	20	560.29 ± 8.74 ^d^	556.20	564.38	
	PXC	20	365.67 ± 19.11 ^a^	356.73	374.62	
BFC	GIC	20	237.68 ± 9.37 ^b^	233.30	242.07	<0.001
	RMGIC	20	454.90 ± 7.95 ^c^	451.18	458.62	
	SARC	20	657.72 ± 20.60 ^d^	648.07	667.36	
	PXC	20	131.26 ± 5.37 ^a^	128.75	133.78	

Abbreviations used in this table: SSC = stainless steel crown; BFC = BioFlx crown; GIC = glass ionomer cement; RMGIC = resin-modified glass ionomer cement; SARC = self-adhesive resin cement; PXC = zinc polycarboxylate cement. * Welch’s analysis of variance test showed statistically significant differences between groups (*p*-value < 0.05). The Dunnett T3 test showed statistically significant differences between groups with similar small letters (a < b < c < d).

**Table 4:** The CFPs among different types of luting cement groups within each crown.

Crown	Cement	Frequency	CFP Score			
			1	2	3	4
SSC	GIC	n	13	7	0	0
		%	65%	35%	0%	0%
	RMGIC	n	17	3	0	0
		%	85%	15%	0%	0%
	SARC	n	18	2	0	0
		%	90%	10%	0%	0%
	PXC	n	0	0	5	15
		%	0%	0%	25%	75%
BFC	GIC	n	0	0	16	4
		%	0%	0%	80%	20%
	RMGIC	n	0	0	7	13
		%	0%	0%	35%	65%
	SARC	n	0	0	2	18
		%	0%	0%	10%	90%
	PXC	n	4	16	0	0
		%	20%	80%	0%	0%

Abbreviations used in this table: SSC = stainless steel crown; BFC = BioFlx crown; CFP = cement failure pattern; GIC = glass ionomer cement; RMGIC = resin-modified glass ionomer cement; SARC = self-adhesive resin cement; PXC = zinc polycarboxylate cement.

## Data Availability

The original contributions presented in this study are included in the article/Supplementary Materials. Further inquiries can be directed to the corresponding author.

## Supplementary Materials

The following supporting information can be downloaded at https://www.mdpi.com/article/10.3390/ma18061287/s1. Table S1: Materials used in this study; Table S2: Comparison Between 3M^TM^ stainless steel crowns and NuSmile^®^ BioFlx^®^ crowns; Figure S1: (a) SSC specimens’ preparation; (b) BFC specimens’ preparation; (c) assessment of CFP after pullout test; Figure S2: Experimental flow chart for the study.
