# Supplementary material for: Retention of Pediatric BioFlx Crowns Versus Stainless Steel Crowns Using Different Types of Luting Cements: An In Vitro Study
Source: Materials (Basel). 2025 Mar 14;18(6):1287. doi: 10.3390/ma18061287 (PMC11943875; doi:10.3390/ma18061287)

Supplemental Tables and Figures:

Table S1: Comparison Between 3M™ Stainless Steel Crowns (SSCs) and NuSmile® BioFlx® Crowns (BFCs).

Feature	3M™ Stainless Steel Molar Crowns	NuSmile® BioFlx® Molar Crowns
Material	Nickel-chromium stainless steel	Hybrid resin polymer, Bis-GMA free, contain no metal
Pre-formed Design	Pre-belled, pre-crimped, and pre-festooned for minimal trimming and contouring.	Pre-contoured and pre-crimped
Aesthetic Appearance	Poor (Metallic)	Excellent (Tooth-colored)
Flexibility	Rigid	Flexible
Fit	Snug/active fit	Same of SSCs
Wear Resistance	High	Same/better than SSCs
Gingival Health	Good	Excellent
Preparation	Requires minimal tooth preparation	Same of SSCs
Indication	Posterior teeth	Anterior and Posterior teeth
Contraindications	- Metal allergies (nickel sensitivity)<br>- Esthetic concerns	- Hall Technique<br>- Manipulation with crimping<br>- Bruxism

Table S2: Materials used in this study.

Materials	Manufacturing company
BioFlx crowns (size 4)	NuSmile® Inc., Houston, TX; USA
Stainless steel crowns (size 4)	3M™ ESPE, St. Paul, MN, USA
Type IV dental die stone	GC Fujirock® EP, Leuven, Belgium
Silicone mold	Deguform® Plus, Dentsply International, Hanau, Germany
Interacrylic Ortho Resin	Interacrylic Ortho, Interdent, Celje, Slovenia
Glass ionomer cement	3M™ Ketac™ Cem Aplicap™; 3M ESPE, Seefeld, Germany
Resin-modified glass ionomer cement	RelyX™ Luting 2; 3M ESPE, St. Paul, Minn, USA
Self-adhesive resin cement	RelyX™ U200; 3M ESPE, Seefeld, Germany
Zinc polycarboxylate cement	Poly-F®; Dentsply, Konstanz, Germany

Figure S1. Experimental flow chart for the study. (clearer in the PDF attachment)

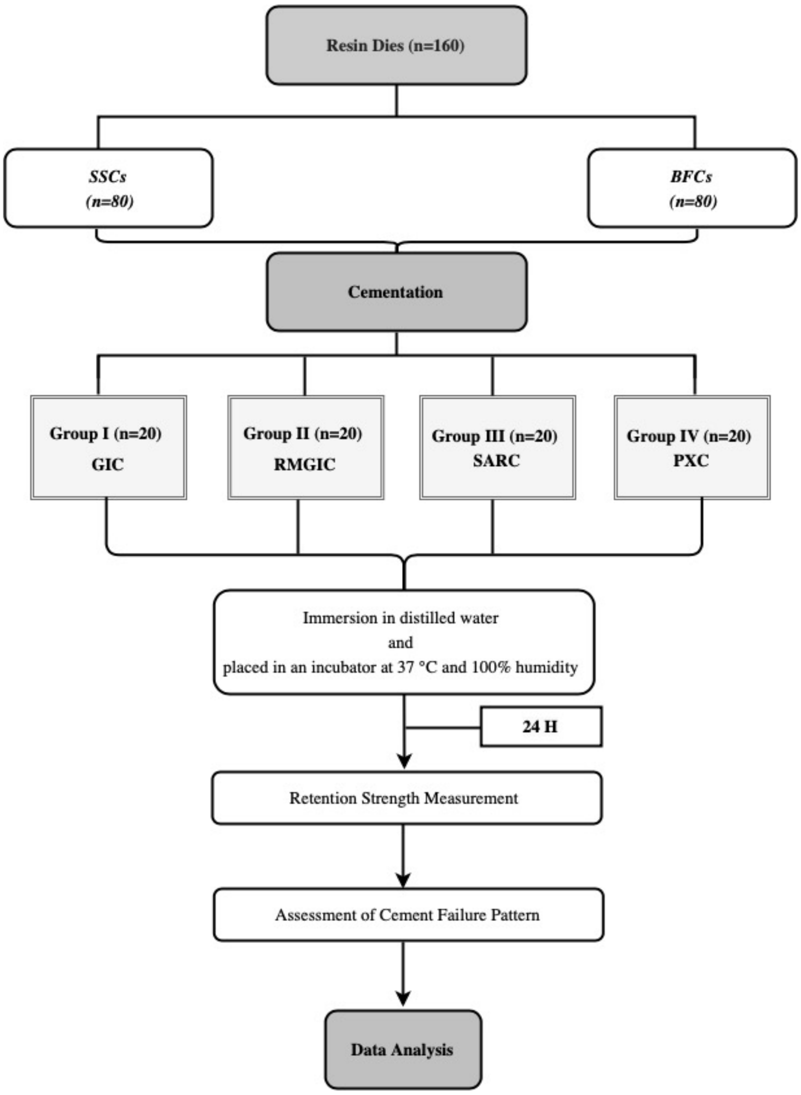

Figure S2. (a) SSC specimens' preparation; (b) BFC specimens' preparation; (c) Assessment of CFP after pull-out test.

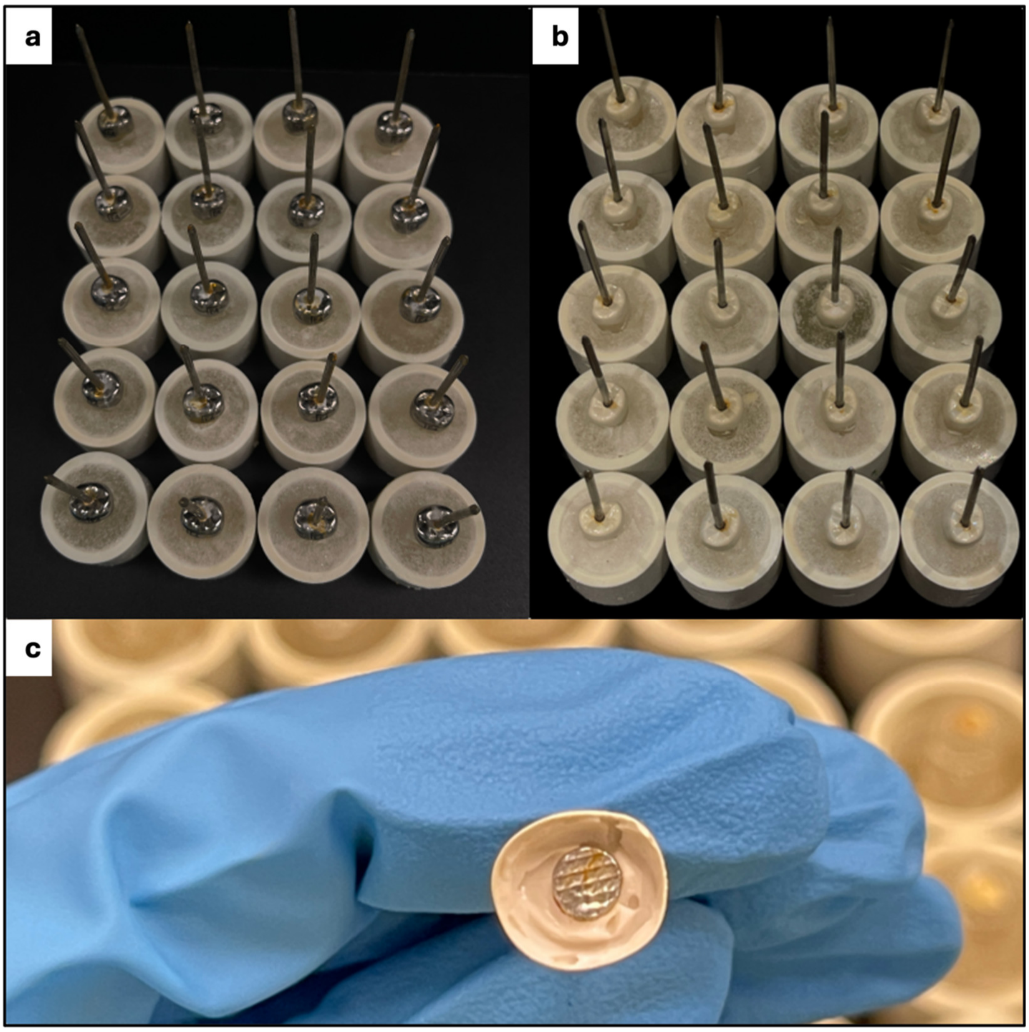

Supplement: Supplementary file 1 [file materials-18-01287-s001.zip › Supplemental Tables and Figures.pdf]
